# FGF4 alleviates the lung cell damage caused by high glucose via AMPK-PGC-1 signaling axis *in vitro* and *in vivo*

**DOI:** 10.3892/ijmm.2025.5710

**Published:** 2025-12-05

**Authors:** Qiujuan Fu, Yongfang Ou, Qin Wu, Jue Gong, Feixia Li, Tuxing Wang, Zhitai Lin, Kejie Huang, Jianlong Xie

**Affiliations:** 1 Pathological Diagnosis Center, Affiliated Hospital of Guangdong Medical University, Zhanjiang, Guangdong 524000, P.R. China; 2 Department of Thoracic Surgery, Affiliated Hospital of Guangdong Medical University, Zhanjiang, Guangdong 524000, P.R. China; 3 First Clinical College, Guangdong Medical University, Zhanjiang, Guangdong 524000, P.R. China

**Keywords:** high sugar, diabetes, fibroblast growth factor 4, inflammation, oxidative stress

## Abstract

Long-term hyperglycemia can damage the capillaries and neural regulation of the lungs, leading to pulmonary microvascular disease and neural regulation disorders, causing abnormalities in lung structure and function. The present study explored the effect of fibroblast growth factor (FGF)4 as a potential therapeutic growth factor on the effect of hyperglycemia on the lungs *in vitro* and *in vivo* models. The effect of FGF4 on the damage of lung cells caused by high glucose was evaluated *in vitro* and *in vivo* by a series of biochemical experiments (indirect immunofluorescence, western blotting, immunohistochemistry and siRNA). The results showed that FGF4 could effectively alleviate the inhibition of lung cell proliferation caused by high glucose. Further experiments found that high glucose caused inflammation, oxidative stress and fibrosis of lung cells, while the above pathological reactions were alleviated after treatment with FGF4. Further mechanism research showed that FGF4 treatment could markedly improve the survival rate of lung cells, reduce cell death and inflammatory responses and enhance the antioxidant stress resistance of cells. These effects are achieved by activating the adenosine monophosphate (AMP)-activated protein kinase (AMPK)-peroxisome proliferator-activated receptor coactivator 1 (PGC-1) signaling axis, which plays an important role in regulating cellular metabolism, antioxidant stress and anti-inflammatory responses. *In vivo* experiments further confirmed the mitigating effect of FGF4 on lung tissue damage caused by high glucose. FGF4 treatment to diabetic model animals, lung function can be markedly improved and the degree of lung inflammation and fibrosis can be reduced. In summary, FGF4 exhibits a significant mitigating effect on high-glucose-induced lung cell damage through the AMPK-PGC-1 signaling axis, providing a new strategy for the treatment of diabetes and its pulmonary complications.

## Introduction

Diabetes is a chronic and progressive metabolic disorder, with an incidence rate increasing year by year (1). Diabetes can cause systemic changes, which increase the mortality and disability rates of patients with diabetes and it has attracted widespread attention from clinicians and patients (2). However, once diabetes occurs, the symptoms are generally severe and progress rapidly, which seriously threatens the survival of patients with diabetes (3). Diabetes complications have always been a research hotspot and focus in the diagnosis and treatment for researchers, including neurological complications, microvascular complications and macrovascular complications. Macrovascular complications mainly include cardiovascular and cerebrovascular complications caused by involvement of the coronary arteries, cerebrovascular vessels and peripheral arteries, as well as diabetic foot. Microvascular complications mainly include diabetic nephropathy and retinopathy (4).

At present, numerous studies focus on diabetes-related lung diseases, especially diabetes-induced lung injury. Due to the abundant reserve of the pulmonary capillary network, the respiratory symptoms of diabetes are not obvious in the early stages of the disease and are easily confused with the manifestations of cardiovascular and cerebrovascular complications, making them easily overlooked by clinicians and patients. However, once diabetes-related lung injury occurs, the symptoms are generally severe and progress rapidly, posing a serious threat to the survival of patients with diabetes (5). Diabetes-related lung injury is mainly manifested as interstitial lung disease, including inflammatory cell infiltration, increased collagen content, oxidative stress, widened alveolar septa and tissue fibrosis (6). Research shows that significant abnormalities in lung function have been found in patients with both type 1 and type 2 diabetes, as well as in children and adults with diabetes (7). Studies have found that the reduced pulmonary diffusion function in patients with diabetes is mainly due to thickening of the alveolar basement membrane and alveolar microvascular disease. Although pulmonary dysfunction is widely observed in patients with diabetes, its specific mechanisms and clinical implications require further exploration (8,9). Diabetes-related pulmonary complications encompass heightened susceptibility to respiratory infections and chronic conditions, including pneumonia, asthma, pulmonary fibrosis and tuberculosis, along with sleep-disordered breathing. A study indicates that >50% of mortality cases among Japanese patients with diabetes stem from these respiratory disorders (9). Another study shows that the fibroblast growth factor (FGF) family is closely related to the biological function of the lungs (10). Other members of the FGF family play important roles in lung development and disease. For example, FGF9 and FGF18 also exhibit important biological effects in idiopathic pulmonary fibrosis (10). Endocrine members of the FGF family, such as FGF19 and FGF21, play an anti-fibrotic role in various organs. Research has shown that FGF19 can markedly reduce pulmonary fibrosis in mouse models and may affect the fibrosis process in the lungs by regulating the function of fibroblasts (11). In addition, the FGF signaling pathway also plays an important role in the repair and regeneration of the lungs. Recent studies have shown that FGF4 has an important biological role in the lungs (12,13). FGF4 belongs to the paracrine subfamily of fibroblast growth factors (which also includes FGF5 and FGF6). It has a molecular weight of 22 kDa and is encoded by a gene located on human chromosome 11 (11q13.3). FGF4 primarily binds to and activates specific fibroblast growth factor receptors (FGFRs), including FGFR1c, FGFR2c and FGFR3c. *In vivo*, FGF4 can be secreted by multiple organs under specific physiological conditions, such as the liver, heart and testes. It has been reported that FGF4 can alleviate diabetes and hyperglycemia (14,15). However, up to now, the effect of FGF4 on diabetic lung injury has not been elucidated.

The present study evaluated the effect of FGF4 on high glucose-induced lung injury. *In vitro*, MLE-12 (a murine alveolar epithelial cell line) and BEAS-2B (a human bronchial epithelial cell line) were used to evaluate FGF4's protective effect against lung cell damage induced by high glucose. It was found that FGF4 markedly alleviated the damage caused by high glucose to lung cells. The current study has laid a solid foundation for further research on the effect of FGF4 on high-glucose lung injury.

## Materials and methods

### Reagents and antibodies

Streptozotocin (STZ) was purchased from MilliporeSigma. PVDF membrane, Immobilon, BCA Kit (protein concentration determination) and hematoxylin-eosin (H&E) staining kit were from Beijing Solarbio Science & Technology Co., Ltd. and Masson trichrome staining solution were from Beijing Solarbio Science & Technology Co., Ltd. Antibodies for TNF-α, IL-6, superoxide dismutase (SOD)1, 4-Hydroxynonenal and NFE2-related factor 2 (Nrf2) were from Abcam. Goat anti-rabbit IgG labeled with horseradish peroxidase, goat anti-mouse IgG labeled with horseradish peroxidase and electrochemiluminescence (ECL) were from Beijing Solarbio Science & Technology Co., Ltd. TGF-β1 and 4-hydroxynonenal (4HNE) were from Cell Signaling Technology, Inc. Cell culture plates, FBS, BSA and TBST buffer were from Beyotime Institute of Biotechnology. CyclinD1, proliferating cell nuclear antigen (PCNA), phosphorylated (p-)IκB, p-P65 and p-STAT3 were from Proteintech Group, Inc. malondialdehyde (MDA; cat. no. A003-1-2), SOD (cat. no. A001-3-2) and glutathione peroxidase (GPX; cat. no. H545-1-1) were from Nanjing Jiancheng Bioengineering Institute.

### Cell culture

MLE12 cells and BEAS-2B were cultured using Dulbecco's modified Eagle's medium (DMEM; Thermo Fisher Scientific, Inc.) supplemented with 10% fetal bovine serum (Gibco; Thermo Fisher Scientific, Inc.). The cells were cultured in a 5% CO_2_ incubator at 37°C for 24-48 h.

### CCK-8 detection assay

Cells in the logarithmic growth phase (8,000 per well) were inoculated into 96-well plates. When the cells reached ~70% confluence, they were treated with high glucose (30 mmol/l) at different time points. After incubation for 24 h at 37°C, the absorbance was detected using a microplate reader (OD:450 nm).

### ELISA assay

The cell culture supernatant was collected, subjected to centrifugation (2,000 × g) for 20 min at 4°C then the supernatant was collected for subsequent detection. The reagent kit was taken out of the refrigerator and allowed to reach room temperature. The standard curve was prepared according to the kit's operating instructions (TNF-α, cat. no. SEKM-0034; IL6, cat. no. SEKM-0007; IL-1β, cat. no. SEKM-0002) and the expression levels of inflammatory factors were finally calculated based on the results of the microplate reader.

### Cellular immunofluorescence assay

Lung cells were seeded onto 12-well cell culture plates containing coverslips (5×10^4^). After seeding, the plates were transferred to a cell incubator until the cells reached 60% confluence. After FGF4 administration, the seeded cell culture plates were removed from the incubator and the culture supernatant was gently aspirated from each well to remove metabolites and unabsorbed nutrients. Each well was washed to ensure removal of non-cellular components. Fixative (4% paraformaldehyde solution) was slowly and evenly added to ensure coverage of all cells for 20 min at 37°C. After fixation, the fixative was carefully discarded to avoid damaging the cells. Each well was washed four times to remove excess fixative. For cell permeabilization, Triton X-100 was appropriately diluted with PBS to 0.5%. This permeabilization solution was added at 200 *μ*l per well to cover the cells and incubated at room temperature for 10 min. After washing with PBS, 10% goat serum solution (cat. no. S9070; Beijing Solarbio Science & Technology Co., Ltd.) was added at 200 *μ*l per well and incubated at room temperature for 1 h to block non-specific sites in the wells and reduce non-specific binding of subsequent primary antibodies to non-target proteins. After blocking, the corresponding primary antibodies (LC3, 1:50 dilution; LAMP1, 1:60 dilution; TOM20, 1:100 dilution) were added and incubated overnight at 4°C. After incubation, 1 ml of PBS was added to each well and unbound primary antibodies were removed by washing to reduce background signal. Diluted secondary antibody solution (1:500 dilution) was added for 60 min to promote the formation of complexes between the secondary antibody and the primary antibody-bound protein. After secondary antibody incubation, the wells were washed four times with PBS. DAPI was used to counterstain the cell nuclei. After secondary antibody incubation and washing, 200 *μ*l of DAPI solution was added to label the cell nuclei through the binding of DAPI (0.5 *μ*g/ml) to DNA for 24 h at 37°C. After DAPI incubation, the cells were washed with PBS in the dark at low speed. After mounting, the cell samples were observed using a confocal microscope (Fv3000; Olympus Corporation).

### Measurement of mitochondrial membrane potential (MMP)

MMP was assessed using the fluorescent dye TMRE. Cells were seeded onto confocal dishes and incubated with 2 *μ*g/ml TMRE at 37°C in a 5% CO_2_ incubator for 30 min. After washing, the cells were examined under a confocal microscope (Fv3000; Olympus Corporation).

### JC-1 staining

After treatment with FGF4, the cells were washed three times with PBS. JC-1 staining working solution was added to the cells and incubated in a culture incubator for 30 min at 4°C. Upon completion of the incubation, the cells were washed three times with PBS and the cell samples were then examined using a (Fv3000; Olympus Corporation).

### Determination of oxidative stress indicators

The determination of MDA (cat. no. A003-1-2), SOD (cat. no. A001-3-2) and GPX (cat. no. H545-1-1) was performed using commercial kits according to the manufacturer's instructions. The cell supernatant was collected and the absorbance of the sample was measured at a 450 nm wavelength.

### Knockdown of adenosine monophosphate (AMP)-activated protein kinase (AMPK) by siRNA

The AMPK-targeting siRNA was designed and synthesized by Shanghai GenePharma Corporation with the following sequences: Sense strand 5'-CAGGCAUCCUCAUAAUUTT-3' and Anti-sense strand 5'-AAUUAUAUGAGGAUGCCUGTT-3'. A scrambled control siRNA (Sense: 5'-UUCUCCGAACGUGUCACGUdTdT-3'; Anti-sense: 5'-ACGUGACACGUUCGGAGAAdTdT-3') was used as negative control. For transfection, 15 pmol of siRNA was pre-mixed with 1.5 *μ*l Lipofectamine^®^ 3000 (Invitrogen; Thermo Fisher Scientific, Inc.) for 5 min before being added to cells. After 8 h of incubation at 37°C, the medium was replaced with fresh complete medium, followed by 24-48 h of culture before assessing transfection efficiency (Fig. S1).

### Establishment of diabetic mouse models

Male C57BL/6J mice (n=20; weight: 20-22g; 6-8 weeks old) were purchased from Guangdong Yaokang Biotechnology Co., Ltd. and housed at an indoor temperature of 21±2°C with a humidity of 30-70% (12-h light/dark cycle). The animals had *ad libitum* access to food and water. After one week of adaptive feeding, all experimental mice were subjected to body weight and fasting blood glucose measurements. A type 1 diabetic model was established using a low-dose STZ intraperitoneal injection method as reported in the literature. A certain amount of STZ powder was dissolved in 0.1 M sodium citrate solution (pH 4.5) to prepare a 1% STZ solution. Prior to administration, mice in all groups were fasted for 3 h with free access to water. Mice in the diabetes mellitus (DM) groups received intraperitoneal injections of STZ at a dose of 50 mg/kg body weight once daily for five consecutive days, while mice in the Ctrl groups received intraperitoneal injections of an equal volume of 0.1 M sodium citrate solution once daily for five consecutive days. One week after the last STZ injection, blood glucose levels were measured from tail vein blood samples. Mice with blood glucose levels ≥16.7 mmol/l on three separate days were considered to have successfully developed diabetes. Mice that did not meet this criterion received supplementary injections until their blood glucose levels reached the required threshold. After successfully establishing the DM model, the mice were randomly divided into four groups (n=5 per group): The control group, DM group, DM + FGF4 treatment group (0.5 mg/kg) and DM + FGF4 treatment group (1 mg/kg). FGF4 was administered via intraperitoneal injection at a dose of 0.5-1 mg/kg twice a week for a total experimental period of 15 days. At the end of the experiment, all mice were sacrificed by anesthesia (the mice were sacrificed before the end of the experiment due to reaching humane endpoint criteria) to ensure no additional suffering. The criteria (humane endpoints) established in this study also included emergency situations that might occur during the experimental process, including: 20% body weight loss from baseline, severe mobility impairment, overt signs of distress and end-stage disease. The entire experimental period was 45 days. Animal health and behavior were monitored twice daily (morning and evening) throughout the study (physical condition, behavioral signs). Sacrifice was performed by cervical dislocation, under anesthesia (sodium pentobarbital; 50 mg/kg, intraperitoneal injection). Mortality was confirmed by sustained absence of vital signs (respiration, heartbeat, reflexes) for 1 min. The present study was performed in accordance with the NIH Guide for the Care and Use of Laboratory Animals and approved by the Institutional Animal Care and Use Committee (IACUC) of Guangdong University Affiliated Hospital (202410-235) (https://www.gdmuah.com/kxyj/llsc.htm).

### H&E staining

After fixation with 4% formaldehyde for 24 h at 25°C, tissue samples were dehydrated using alcohol of different concentrations. The tissues were embedded in paraffin and sectioned at 4 *μ*m. The tissue sections were stained with hematoxylin staining solution at room temperature for 5 min. After rinsing with running water, the sections were treated with differentiation solution for 30 sec. After washing, eosin staining solution was added to stain the sections for 30 sec. The sections were immersed in tap water for 5 min. After clearing with xylene, the sections were mounted using neutral balsam. The tissue sections were then observed using a light microscope.

### Masson staining

Lung tissue sections (4 *μ*m) were incubated with Weigert iron hematoxylin staining solution for ~10 min at 25°C. The sections were then treated with alcohol differentiation solution for 5-15 sec. After rinsing with tap water, the sections were immersed in Masson bluing solution for 3 min to restore blue coloration. After rinsing with tap water, the tissue sections were stained with Ponceau fuchsin staining solution for 5-10 min. Subsequently, the sections were rinsed with weak acid working solution for 1 min. They were then washed with phosphomolybdic acid solution for 1-2 min, followed by another rinse with weak acid working solution for 1 min. The tissue sections were directly transferred to aniline blue staining solution for 1-2 min and then rinsed with weak acid working solution for 1 min. After dehydration with ethanol, the sections were observed using a light microscope (magnification, ×10-20). A total of five non-overlapping fields were randomly selected.

### Western blot analysis

Lung tissue was placed in a 2 ml EP tube, pre-cooled tissue RIPA lysis buffer (cat. no. R0020, Solarbio) was added and the tissue was homogenized using a tissue homogenizer. The sample was centrifuged at 13,000 × g for 15 min at 4°C and the supernatant was collected. Protein concentration was measured using a BCA kit. The proteins (30 *μ*g/lane) were separated by 4-10% SDS-PAGE. After electrophoresis, proteins were transferred to a PVDF membrane, which was washed three times with TBST (0.05% Tween) solution for 10 min each. The PVDF membrane with target proteins was blocked in 5% skimmed milk powder at room temperature for 1 h. The membrane was then washed three times with TBST solution for 10 min each. Primary antibodies (PGC-1α, 1:3,000 dilution; TFAM, 1:2,000 dilution; β-actin, 1:4,000 dilution) diluted in 5% BSA were added and the membrane was incubated on a shaker at 4°C overnight. After washing three times with TBST (10 min each), secondary antibodies (HRP-conjugated goat anti-rabbit secondary antibody at a 1:5,000 dilution) were added and incubated for 2 h. ECL luminescent solution was prepared according to the instructions. The PVDF membrane with target proteins was evenly coated with the luminescent solution and placed in a fully automated chemiluminescence imaging system for exposure. Blots were quantified using ImageJ 1.52a software (National Institutes of Health).

### Immunohistochemistry

The tissue sections were permeabilized with 0.1% Triton X-100 for 15 min. The sections (4 *μ*m) were blocked with 10% goat serum (as antigen blocking solution) for 2 h at 25°C. Endogenous peroxidase activity was quenched by incubating the sections with 3% hydrogen peroxide in methanol for 15 min at room temperature. The corresponding primary antibodies solution (IL6, cat. no. 1:EPR23819-103, 1:150 dilution; IL-1β, cat. no. EPR19147, 1:200 dilution) was then added and the slides were incubated at 4°C for 12 h. After incubation, the sections were thoroughly washed with PBS. Fluor594-labeled secondary antibody (cat. no. K1034G, 1:500 dilution) was added and incubated for 60 min at 37°C. After washing, the sample was observed using a laser confocal microscope (Fv3000; Olympus Corporation).

### Statistical analysis

All experimental results are presented as mean ± SD, SPSS 24.0 (IBM Corp.) was used for statistical analysis. All data were tested for normality using the Shapiro-Wilk method and data that met normal distribution were analyzed using One-way ANOVA variance analysis. Tukey's post hoc test was used to assess the associations among different groups. Data that did not meet normal distribution were analyzed using the rank sum test. P<0.05 was considered to indicate a statistically significant difference.

## Results

### The effect of FGF4 on high glucose-induced lung cell injury

**Figure 1:**
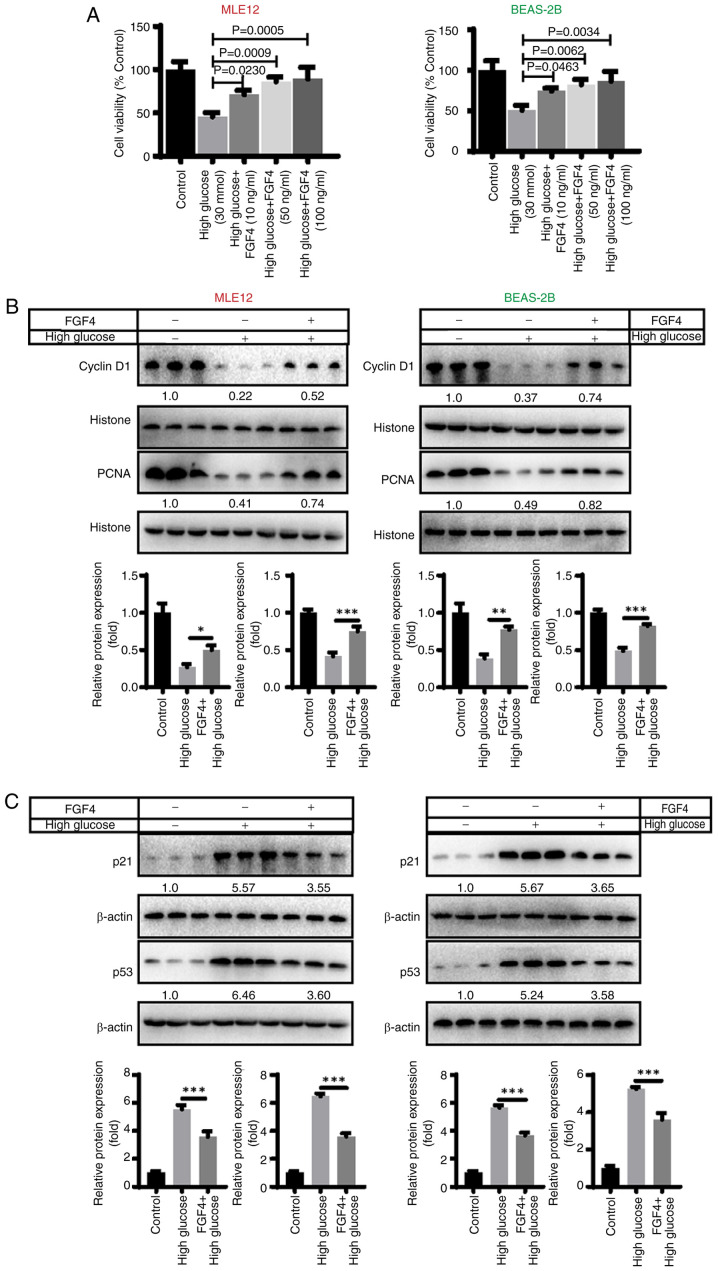
FGF4 alleviated the damage of lung cells caused by hyperglycemia. (A) Cell proliferation is promoted by FGF4 treatment. (B) The effect of FGF4 on the expression of cyclinD1 and PCNA in cells. (C) The effect of FGF4 on the expression of p21 and p53. ^*^P<0.05, ^**^P<0.01, ^***^P<0.001. FGF4, fibroblast growth factor 4; PCNA, proliferating cell nuclear antigen.

CCK8 assays were used to detect the effect of FGF4 on high-glucose-induced lung cells. High glucose treatment [30 mmol/l; this selection of glucose concentrations is based on reference (15)] induced a decrease in the proliferative capacity of lung cells, indicating that high glucose has a significant inhibitory effect on lung cell proliferation. By contrast, when cells were treated with FGF4 (10, 50 and 100 ng/ml; the selection of these concentrations was based on preliminary experiments), the proliferative capacity of cells was markedly increased (Fig. 1A). In addition, the present study evaluated the effect of FGF4 on the expression of cell cycle-related proteins. The results illustrated that the expression of CyclinD1 and PCNA was markedly downregulated in the high glucose group, while in the FGF4 treatment group, the expression level of CyclinD1 was markedly upregulated (Fig. 1B). Similarly, the expression of p21 and p53 (cell cycle inhibitors) was markedly reduced after FGF4 treatment, implying that FGF4 can promote cell proliferation (Fig. 1C).

### The effect of FGF4 on high glucose-induced lung cell inflammation and oxidative stress

**Figure 2:**
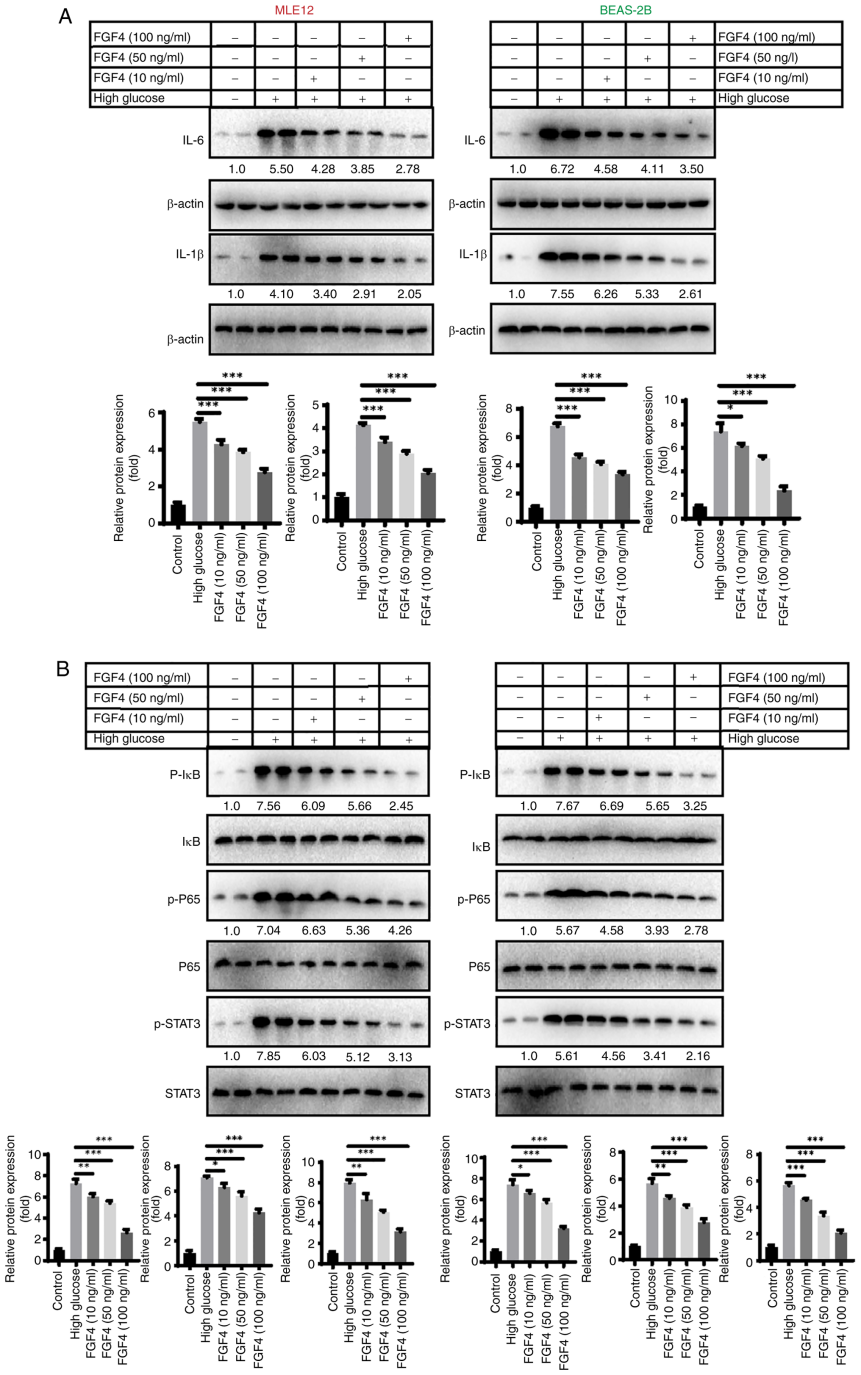
The effect of FGF4 on high glucose-induced inflammation and oxidative stress in lung cells. (A) The effect of FGF4 on IL-6 and IL-1β by western-blot analysis. (B) The effect of FGF4 on the expression levels of p-IκB, p-P65 and p-STAT3. ^*^P<0.05, ^**^P<0.01, ^***^P<0.001. FGF4, fibroblast growth factor 4; p-, phosphorylated.

The present study first evaluated the effect of FGF4 on the expression of inflammatory factors in high glucose-induced lung cells. As shown in Fig. 2A, western blot analysis revealed that treatment with FGF4 at concentrations of 10, 50 and 100 ng/ml led to decreased expression levels of IL-6 and IL-1β. Additionally, the protein expression levels of p-IκB, p-P65 and p-STAT3 were all reduced following FGF4 treatment (Fig. 2B). The present study also assessed the effect of FGF4 on oxidative stress and the results demonstrated that FGF4 could reduce the levels of reactive oxygen species (ROS) and MDA, while increasing the level of SOD (Fig. S2).

### Effect of FGF4 on high glucose-induced lung tissue fibrosis

**Figure 3:**
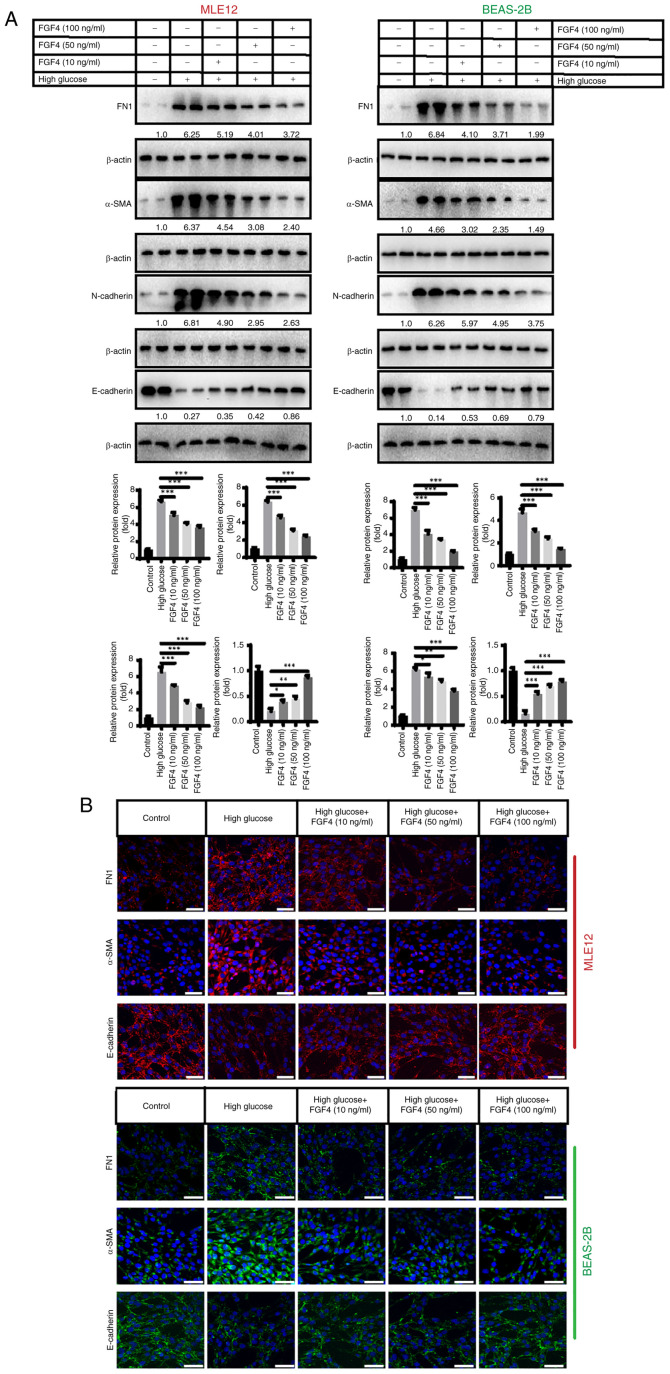
The effect of FGF4 on high glucose-induced lung fibrosis. (A) The effect of FGF4 on the expression of FN1, α-SMA, E-cadherin and N-cadherin. (B) Indirect immunofluorescence analysis of the effect of FGF4 on fibrosis. Scale bar, 50 *μ*m. ^*^P<0.05, ^**^P<0.01, ^***^P<0.001. FGF4, fibroblast growth factor 4; FN1, fibronectin 1; α-SMA, α-smooth muscle actin.

Fig. 3 shows that high glucose stimulation promotes lung tissue fibrosis. The present study investigated the expression of fibronectin 1 (FN1), α-smooth muscle actin (α-SMA) and epithelial cell markers (E-cadherin and N-cadherin). The results revealed that in the high-glucose group, the protein expression of FN1, α-SMA and N-cadherin was upregulated, while E-cadherin protein expression was downregulated. It was observed that after FGF4 treatment, the expression levels of FN1, α-SMA and N-cadherin were also markedly decreased, while E-cadherin expression was increased (Fig. 3A). In addition, indirect immunofluorescence assays yielded similar results (Fig. 3B).

### FGF4 alleviates high glucose-induced inhibition of mitochondrial autophagy

**Figure 4:**
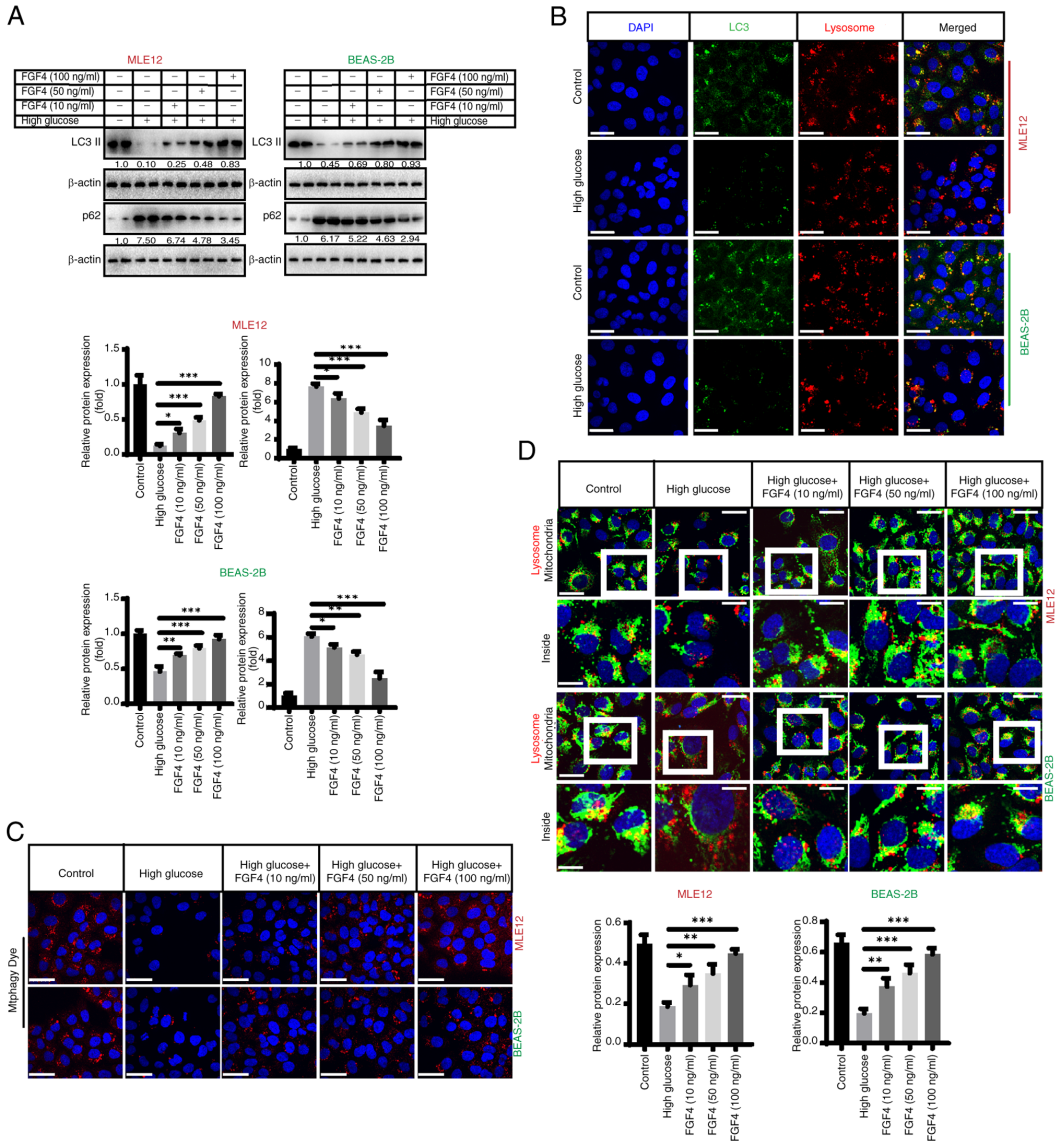
FGF4 alleviates the inhibition of mitochondrial autophagy caused by high glucose. (A) Evaluation of the level of autophagy in the presence of high glucose. (B) High glucose inhibited the autophagy. (C) FGF4 promoted the autophagy through using specific fluorescent probe. (D) Co-localization also showed that autophagy of mitochondria was partially restored under treatment with FGF4. ^*^P<0.05, ^**^P<0.01, ^***^P<0.001. FGF4, fibroblast growth factor 4; PINK1, PTEN-induced kinase 1.

The present study assessed the impact of high glucose on mitochondrial autophagy. It first evaluated the effect of high glucose on autophagy and found that the expression of LC3-II decreased, while the expression level of p62 markedly increased (Fig. 4A). Indirect immunofluorescence also demonstrated that high glucose inhibited autophagy, as the co-localization (yellow fluorescence) of LC3 (green fluorescence) and lysosomes (red fluorescence) was markedly reduced (Fig. 4B). Furthermore, we found that mitophagy (red fluorescence) was markedly inhibited by high glucose treatment and FGF4 promoted autophagy when assessed using specific fluorescent probes (Fig. 4C). Additionally, fluorescence co-localization (yellow fluorescence) showed that mitochondrial autophagy (lysosomes: red fluorescence; mitochondria: green fluorescence) was partially restored following FGF4 treatment (Fig. 4D). In addition, the expression level of TOM20 increased in the high-glucose treatment group, while its level markedly decreased after FGF4 treatment, indicating enhanced mitophagy (Fig. S4A). MMP staining with JC-1 revealed that mitochondrial membrane potential had undergone depolarization and its level increased after FGF4 treatment (Fig. S4B).

In general, PTEN-induced kinase 1 (PINK1)/Parkin mediates autophagy of mitochondria with impaired membrane potential. However, PINK1/Parkin decreased under high-glucose treatment. After FGF4 treatment, the level of mitochondrial autophagy was partially restored (Fig. S4C). This suggested that FGF4 may regulate mitochondrial activity by modulating mitochondrial biosynthesis and autophagy pathways.

### High glucose induces release of mtDNA from lung mitochondria and activates cGAS-STING signaling

**Figure 5:**
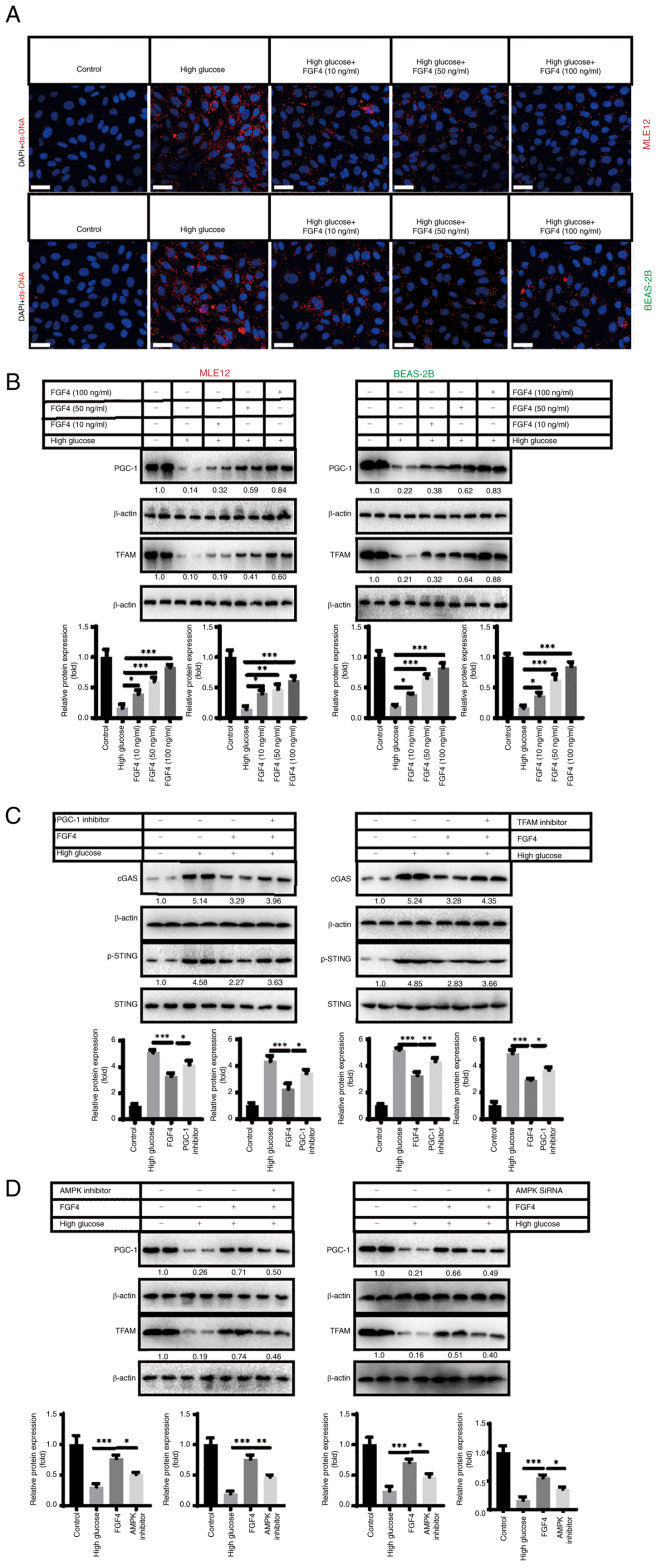
The effect of FGF4 on the cGAS-STING signaling pathway triggered by high glucose. (A) FGF4 inhibited high glucose-promoted the release of mitochondrial DNA into the cytoplasm. Scale bar, 50 *μ*M. (B) FGF4 restored the expression of PGC-1 and TFAM. (C) FGF4 inhibited cGAS-STING signaling. (D) The effect of FGF4 was blocked when AMPK was inhibited. ^*^P<0.05, ^**^P<0.01, ^***^P<0.001. FGF4, fibroblast growth factor 4; PGC-1, activated receptor coactivator 1; TFAM, mitochondrial transcription factor A; AMPK, adenosine monophosphate activated protein kinase.

High glucose-induced injury can lead to mitochondrial damage (16). Therefore, the present study further focused on mitochondria as a target and explored the potential molecular mechanisms by which FGF4 combats high glucose-induced lung cell damage. It was hypothesized that dysfunctional mitochondria may release damage-associated molecular patterns (DAMPs) into the cytoplasm. Mitochondrial DNA (mtDNA), as an important DAMP, further activates the inflammatory response in lung cells. This may be one of the potential molecular mechanisms underlying the current study. To this end, the present study conducted relevant experiments to verify this hypothesis. Using indirect immunofluorescence, it was found that the level of mitochondrial DNA (red fluorescence) in the cytoplasm markedly increased under high-glucose treatment, while mtDNA levels markedly decreased following FGF4 treatment (Fig. 5A). The present study continued to explore how FGF4 repaired mitochondria and reduced mtDNA leakage. A previous study showed that the PGC-1-mitochondrial transcription factor A (TFAM) signaling pathway regulates mitochondrial biosynthesis and maintains mitochondrial function (17). The present study demonstrated that high glucose severely inhibited the expression of PGC-1 and TFAM, while FGF4 treatment partially restored the expression of these molecules (Fig. 5B). When the expression of PGC-1/TFAM was inhibited, it was found that the alleviating effect of FGF4 on cGAS-STING signaling was partly offset (Fig. 5C). Further experiments showed that FGF4 activated the PGC-1/TFAM signaling axis through AMPK: when AMPK was inhibited, the activity of the PGC-1/TFAM signaling pathway was markedly reduced (Figs. 5D and S3). These data suggested that FGF4 regulates cGAS-STING signaling through the AMPK-PGC-1-TFAM signaling axis.

### The effect of FGF4 on body weight and blood glucose in diabetic mice

**Figure 6:**
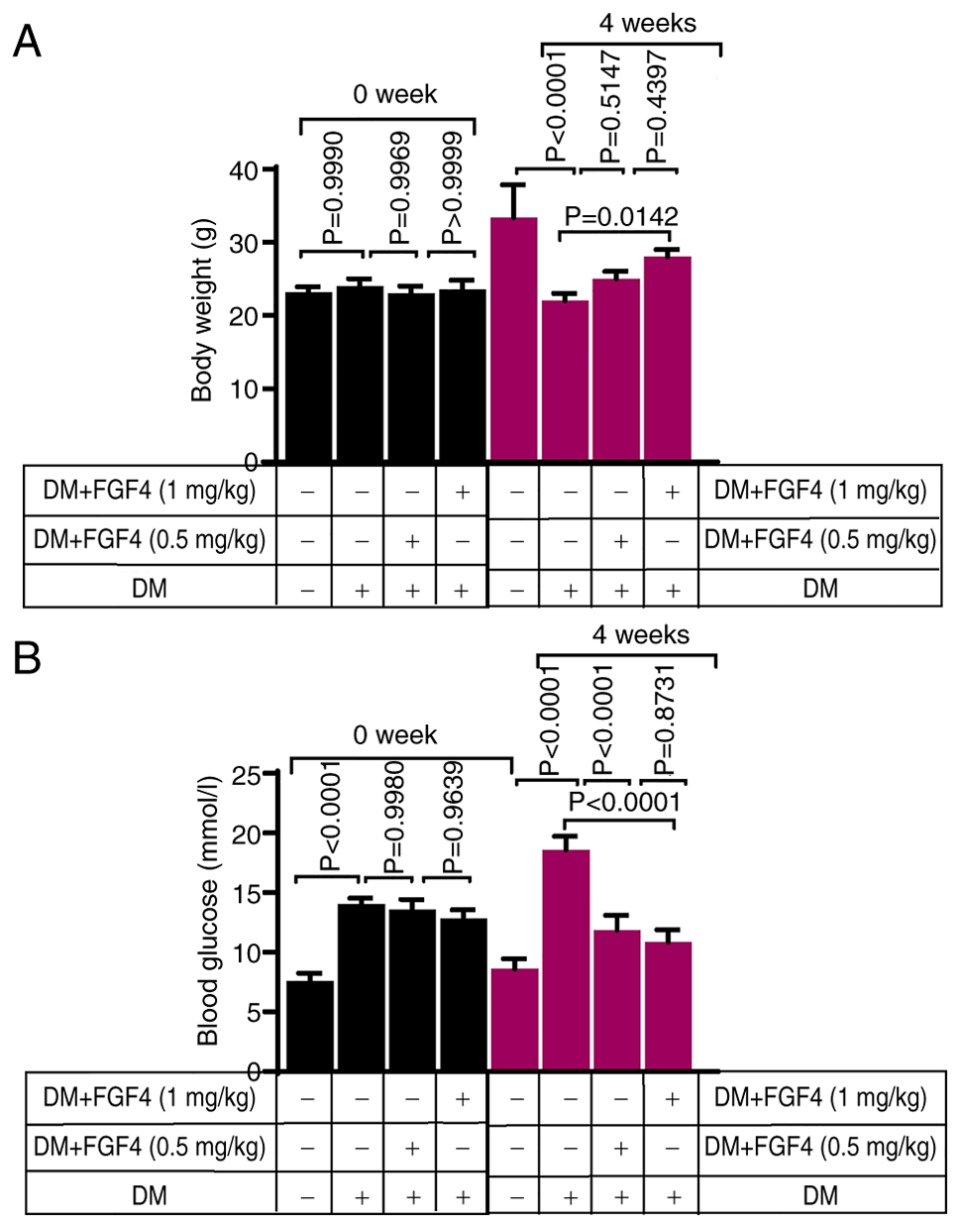
The effect of FGF4 on the body weight and blood glucose of mice. (A) Effect of FGF4 on the body weight of mice. (B) The effect of FGF4 on blood glucose. FGF4, fibroblast growth factor 4; DM, diabetes mellitus.

Changes in body weight of mice in each group before and after the experiment are shown in Fig. 6A. As expected, at week 0, there were no significant differences in body weight among the groups. Compared with the diabetic group, the body weight of mice in the FGF4 treatment group increased slightly, suggesting that FGF4 can alleviate diabetes-induced weight loss. In addition, changes in blood glucose levels in mice across all groups before and after the experiment were evaluated. Blood glucose levels of mice in each group before and after the experiment are shown in Fig. 6B. Prior to the experiment (week 0), blood glucose levels in the diabetic and diabetic + FGF4 groups were markedly higher than those in the control group, indicating successful establishment of the diabetic mouse model. At the end of the fourth week, blood glucose levels in the FGF4 treatment group were markedly lower than those in the diabetic group, suggesting that FGF4 has a significant hypoglycemic effect in diabetic mice, which is consistent with previous findings.

### Effect of FGF4 on lung tissue inflammation in diabetic mice

**Figure 7:**
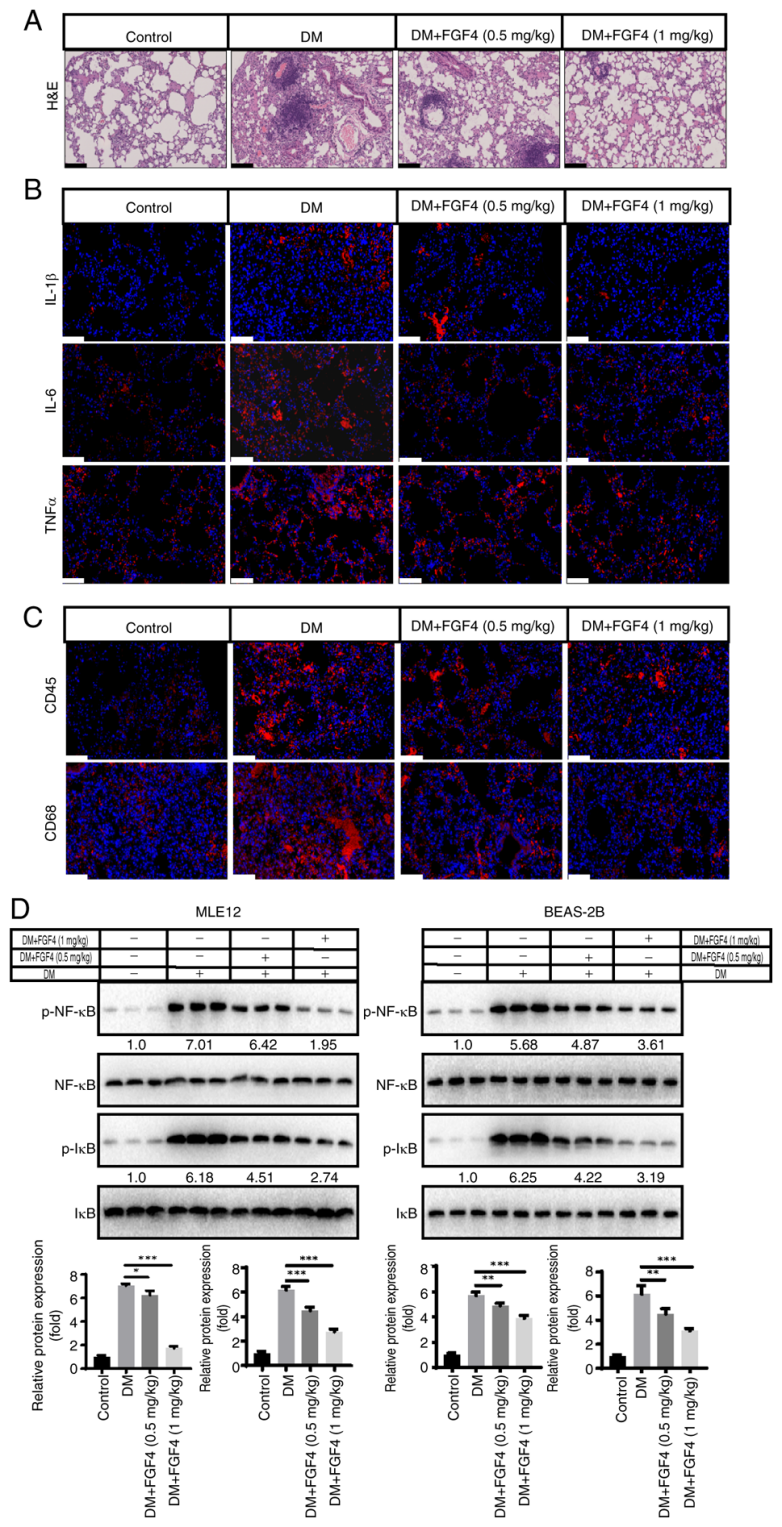
Effect of FGF4 on lung tissue inflammation in diabetic mice. (A) H&E staining analysis of the effect of FGF4 on the lung tissue of diabetic mice. (B) Immunohistochemical analysis of the effect of FGF4 on lung inflammation. Scale bar, 60 *μ*m. (C) FGF4 treatment alleviated the infiltration of inflammatory cells. (D) The effect of FGF4 on the expression of NF-κB. ^*^P<0.05, ^**^P<0.01, ^***^P<0.001. FGF4, fibroblast growth factor 4; H&E, hematoxylin and eosin; DM, diabetes mellitus.

The present study evaluated the effect of FGF4 on pathological changes in lung tissue of diabetic mice. From H&E staining results, compared with the diabetic model group, the alveolar structure in the control group was clear and intact, with no obvious exudation in the alveolar cavity and no significant widening of alveolar septa. However, in diabetic mice, the alveolar structure was destroyed and inflammatory cell infiltration was observed. In the FGF4 treatment group, pathological changes were markedly alleviated compared with the DM group (Fig. 7A). To further evaluate pulmonary inflammation in diabetic mice, we used immunohistochemistry to detect the levels of TNF-α, IL-1β and IL-6. As shown in Fig. 7B, compared with the control group, the expression levels of TNF-α, IL-1β and IL-6 in lung tissues of diabetic mice were markedly higher, while their expression levels in the FGF4 treatment group were markedly lower than those in the diabetic group (DM). These results suggested that FGF4 can downregulate the expression of inflammatory factors. Furthermore, immunofluorescence showed that the infiltration of inflammatory cells (CD45^+^ and CD68^+^ cells) was markedly reduced (Fig. 7C). In addition, the expression level of the inflammatory factor NF-κB was also markedly decreased (Fig. 7D).

### Effect of FGF4 on the degree of pulmonary fibrosis in diabetic mice

**Figure 8:**
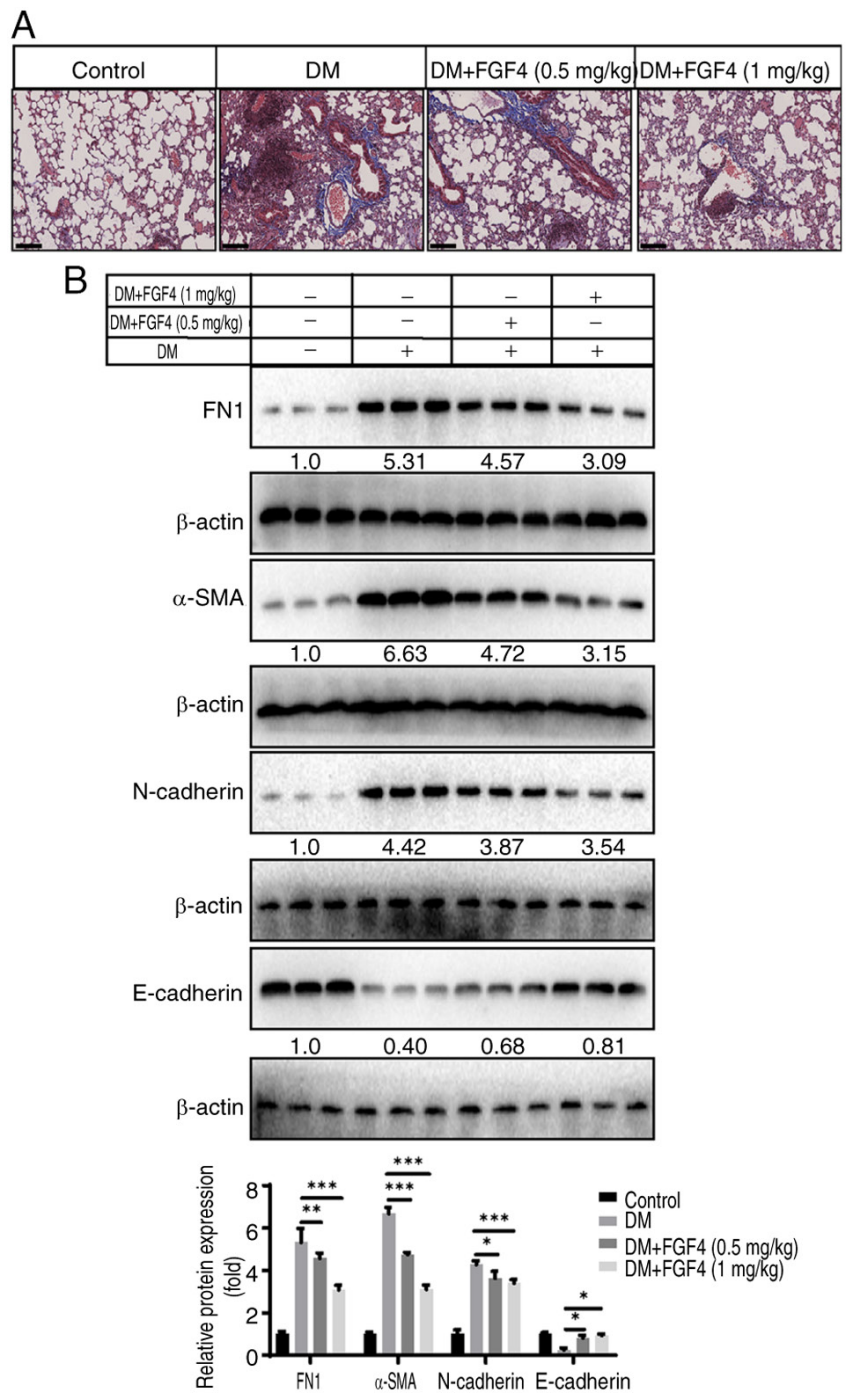
The effect of FGF4 on pulmonary fibrosis in diabetic mice. (A) Masson staining analysis of the effect of FGF4 on lung fibrosis. (B) The effect of FGF4 on marker molecules for lung tissue fibrosis. FGF4, fibroblast growth factor 4; DM, diabetes mellitus. Scale bar, 200 *μ*M. ^*^P<0.05, ^**^P<0.01, ^***^P<0.001.

The present study continued to evaluate the effect of FGF4 on the fibrosis level in mouse lung tissues and performed Masson staining: As shown in Fig. 8A, there was no significant collagen deposition in lung tissues of the control group, while more blue-stained collagen fibers were observed in lung tissues of the DM group. By contrast, only a small amount of collagen fibers were observed in lung tissues of the FGF4 treatment group. The present study also examined the expression of FN1, α-SMA, E-cadherin and N-cadherin proteins. The results showed that after FGF4 treatment, the expression of FN1, α-SMA and N-cadherin was downregulated, while the expression of E-cadherin was upregulated (Fig. 8B).

### Effect of FGF4 on the oxidative-antioxidant balance in lung tissue of diabetic mice

**Figure 9:**
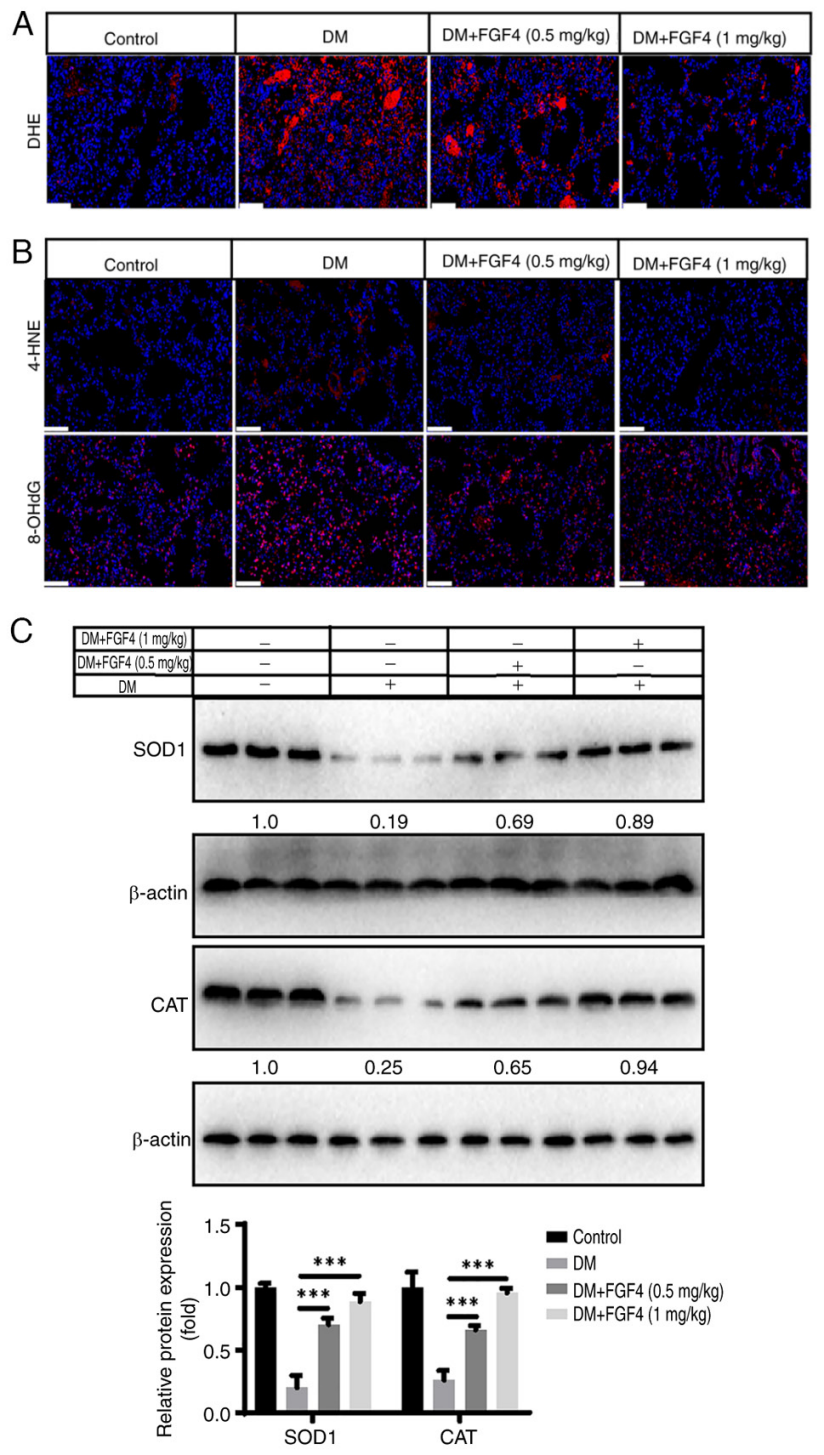
Effect of FGF4 on oxidative stress in lung tissue of diabetic mice. (A) The effect of FGF4 on ROS by DHE. (B) The effect of FGF4 on 4-HNE/8-OHdG (scale bar, 60 *μ*m). (C) The effect of FGF4 on the expression of SOD1 and CAT. ^***^P<0.001. FGF4, fibroblast growth factor 4; ROS, reactive oxygen species; SOD, superoxide dismutase; CAT, catalase; DM, diabetes mellitus.

Given that oxidative stress is involved in the pathogenesis of diabetes-related lung injury (1), the present study first analyzed the effect of FGF4 on oxidative stress. DHE staining showed that ROS levels in lung tissue markedly decreased under FGF4 treatment (Fig. 9A). In addition, the present study evaluated the levels of 4-HNE and 8-OHdG. Compared with the control group, the expression level of 4-NHE and 8-OHdG in the diabetic group was markedly increased, while the expression levels of 4-HNE and 8-OHdG in the FGF4 group were markedly lower than those in the DM group. These results suggested that oxidative stress damage occurs in the lung tissues of diabetic mice and FGF4 administration can alleviate this oxidative stress response (Fig. 9B). Antioxidant proteins, including SOD1 and catalase (CAT) were also evaluated. The results showed that the expression of SOD1 and CAT in the diabetic group was markedly reduced, whereas their expression levels were markedly increased after FGF4 treatment (Fig. 9C).

### Effect of FGF4 on mitochondrial autophagy and cGAS-STING signaling in vivo

**Figure 10:**
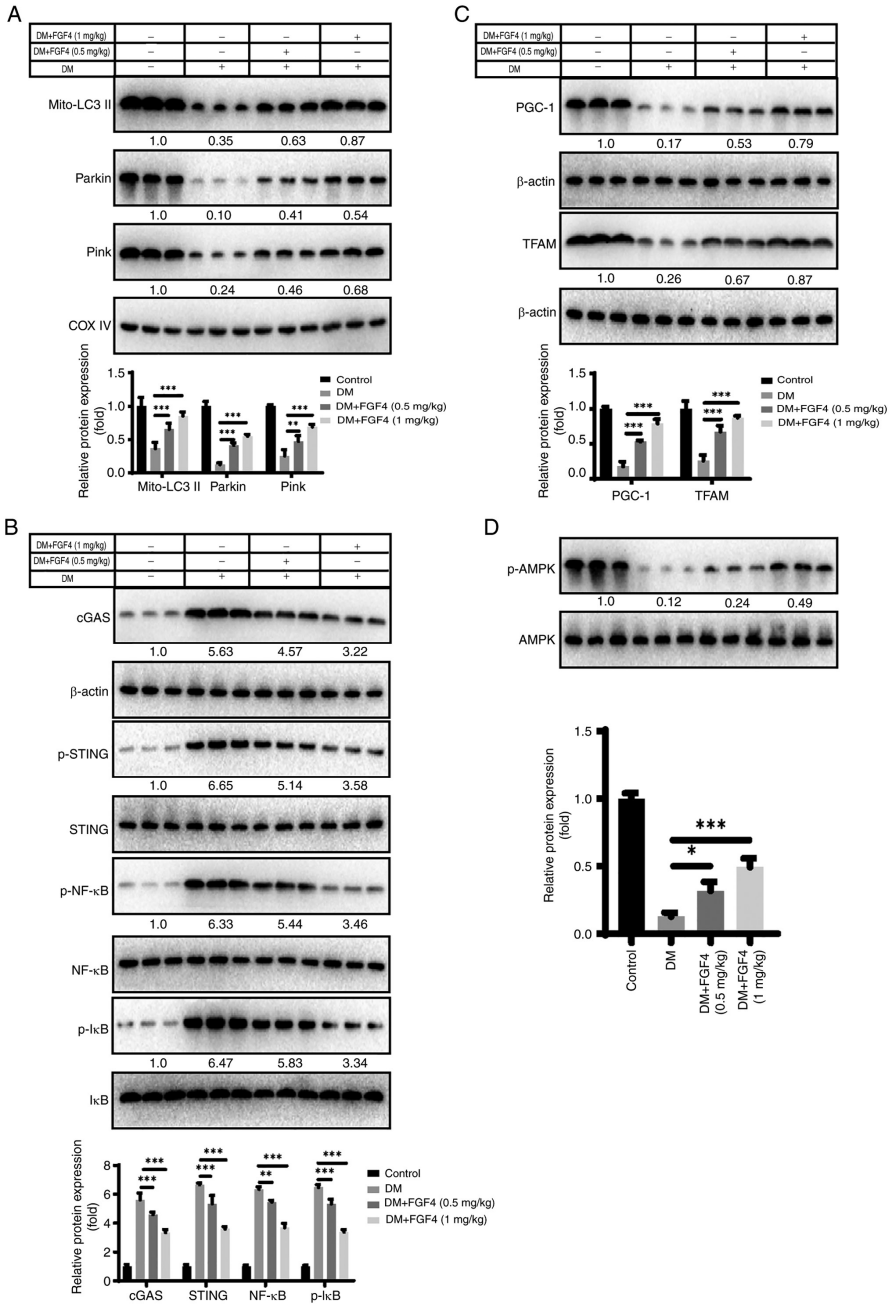
FGF4 improves mitochondrial autophagy and the cGAS-STING signaling pathway. (A) FGF4 alleviated the mitochondrial autophagy by western blotting. (B) FGF4 weakened the cGAS-STING signaling pathway. (C) FGF4 enhanced the expression of PGC-1-TFAM. (D) FGF4 treatment increased AMPK phosphorylation *in vivo*. ^*^P<0.05, ^**^P<0.01, ^***^P<0.001. FGF4, fibroblast growth factor 4; PGC-1, activated receptor coactivator 1; TFAM, mitochondrial transcription factor A; AMPK, adenosine monophosphate activated protein kinase.

The present study also evaluated explored the potential molecular mechanism by which FGF4 ameliorates lung cell injury *in vivo*. First, it analyzed the effect of diabetes mellitus (DM) on mitochondrial autophagy. Western blot analysis showed that FGF4 alleviated the inhibition of mitochondrial autophagy induced by DM (Fig. 10A). The present study further analyzed the effect of FGF4 on the cGAS-STING signaling pathway and the results revealed that the activity levels of the cGAS/STING/NF-κB signaling pathway were markedly reduced (Fig. 10B). Similarly, FGF4 increased the expression of PGC-1 and TFAM, which is consistent with the findings from *in vitro* cell models (Fig. 10C). Additionally, FGF4 treatment increased AMPK phosphorylation *in vivo* (Fig. 10D).

## Discussion

Diabetes can lead to lung injury and abnormal lung function, a condition known as diabetic lung disease. Chronic and persistent hyperglycemia stimulates the production of inflammatory proteins, which accumulate in small blood vessels and connective tissues. Glycation modifications of collagen and elastin in lung tissue can induce interstitial fibrotic changes (18). In addition, diabetes-induced systemic inflammation is another major cause of lung injury. Dysregulation of inflammatory regulatory mechanisms can trigger excessive inflammatory responses in the lungs, resulting in impaired lung function (19). In the present study, a type 1 diabetes model was established via intraperitoneal injection of low-dose STZ. After successful modeling, FGF4 intervention was administered to further investigate the role and potential mechanism of FGF4 in diabetes-related lung injury. Results showed that blood glucose levels in experimental mice decreased and their body weight rebounded. These findings indicated that FGF4 exhibits a protective effect against diabetes-related lung injury in both *in vitro* and *in vivo* models. First, to the best of the authors' knowledge, this is the first study to reveal that FGF4 can alleviate high-glucose-induced lung injury; Second, it identified a new molecular mechanism by which FGF4 promotes mitophagy to mitigate high glucose-mediated lung damage; Third, it was found that FGF4 can ameliorate lung injury, particularly pulmonary fibrosis (another original observation).

*In vitro* experiments showed that FGF4 could mitigate high glucose-induced lung cell damage and promote cell proliferation. For instance, FGF4 increased Ki67 expression while suppressing p21 and p53 expression.

Inflammatory responses and oxidative stress play important roles in the pathogenesis of diabetes-related lung injury (20). The present study first evaluated the effect of FGF4 on lung tissue in both *in vitro* and *in vivo* models of diabetic mice. H&E staining showed that the alveolar structure was destroyed, the alveolar septa were markedly widened and inflammatory cell infiltration was observed in the widened alveolar septa, small bronchi and around blood vessels, suggesting that diabetic mice had developed pulmonary complications. Furthermore, the levels of the inflammatory factors TNF-α, IL-1β and IL-6 were assessed and the results showed that FGF4 treatment reduced the inflammatory response. The present study also evaluated NF-κB levels and found that its expression was markedly decreased.

Clinical and laboratory evidence supports that oxidative stress plays an important role in the pathophysiology of diabetes and its complications. Continuous high-glucose stimulation can lead to the accumulation of large amounts of ROS in the lungs, inducing oxidative stress injury and further aggravating pulmonary inflammatory responses (21). The coexistence of oxidative stress and inflammation is a common cause and high-risk factor for diabetes. The present study found that FGF4 alleviated oxidative stress levels in both *in vitro* and *in vivo* models. Previous studies have also shown that multiple members of the FGF family exhibit antioxidant stress effects; for example, FGF1, FGF2 and FGF21 play important roles in antioxidant stress and aging (10,22).

In addition, pulmonary fibrosis is another important pathological change in diabetes-induced lung injury (23). A key mechanism promoting tissue fibrosis is epithelial-mesenchymal transition (EMT) and animal experiments have confirmed that inhibiting EMT can reduce the progression of tissue fibrosis. Continuous high-glucose stimulation can induce EMT, mainly mediated by the upregulation of fibroblast-specific TGF-β1. The present study showed that FGF4 relieved pulmonary fibrosis in diabetic mice by inhibiting the production of pulmonary fibrosis, as evaluated by the expression of relevant markers.

Autophagy is a cellular degradation program involved in responses to various stimuli (24). Thus, autophagy dysfunction may lead to a number of pathological changes, including cancer and immune abnormalities (25). It is ubiquitous across organisms, involving the formation of autophagosomes that recognize damaged or dysfunctional organelles and macromolecules. Autophagy can be divided into different types, such as chaperone-mediated autophagy, microautophagy and macroautophagy. By analyzing its nature, mitochondrial autophagy (mitophagy) is recognized as a specialized type of autophagy and studies have found that it is closely associated with cellular homeostasis. Based on differences in phagocytic and recognition mechanisms, these processes can be roughly categorized into two pathways: Ubiquitin-dependent and receptor-dependent. In the first pathway, PINK1/Parkin is most critical; its mechanism primarily involves the binding of damaged mitochondria to ubiquitinated outer membrane proteins, which recruits p62. p62 then binds to LC3 on the autophagosome membrane, promoting the encapsulation of damaged mitochondria within autophagosomes to complete mitochondrial degradation (26). The second pathway relies on interactions between γ-aminobutyric acid receptor-related proteins and LC3 on autophagosomal membranes, such as NIX, FUNDC1 and BNIP3 located on the outer membrane of damaged mitochondria. FUNDC1 and BNIP3 directly interact with LC3 before being recognized by phagocytic vesicles (26).

The present study showed that under high-glucose conditions, FGF4 markedly enhanced mitophagy levels. Specifically, LC3 protein expression was markedly increased, while p62 protein levels were markedly reduced. Additionally, under high-glucose conditions, the mitochondrial membrane potential in the FGF4 treatment group was higher than that in both the empty vector group and the high-glucose group. This finding further confirmed the increased mitophagy levels in the FGF4-treated group.

The cGAS-STING signaling pathway and mitochondrial autophagy play important roles in inhibiting mtDNA leakage (27). Mitophagy is a cellular process that maintains mitochondrial health by eliminating damaged mitochondria. When mitochondria are damaged, mitophagy prevents the accumulation of mtDNA in the cytoplasm, thereby avoiding the triggering of inflammatory responses mediated by the cGAS-STING pathway (28). In this process, mitophagy not only optimizes mitochondrial function but also maintains cellular homeostasis by inhibiting the cGAS-STING signaling. By contrast, the cGAS-STING pathway triggers an immune response upon detecting mtDNA leakage, further affecting cellular function and survival. The present study found that FGF4 can inhibit the activation of cGAS-STING by regulating the PGC-1α-TFAM signaling pathway.

It is important to mention that most other FGF family members (such as FGF21 and FGF23) have been widely reported for their association with hyperglycemia, whereas research on FGF4 remains relatively limited. In terms of practical application potential, the experimental results demonstrated that FGF4 holds significant promise, as its biological activity may be stronger compared with other FGF members. Therefore, FGF4 may be a promising drug for treating lung damage caused by diabetes.

The current study has several limitations. First, toxicological studies of FGF4 need to be conducted in future work. Second, the targeting specificity of FGF4 requires improvement, as it can distribute to various tissues and organs after entering the bloodstream. Third, the short half-life of FGF4 needs to be addressed in future studies, to overcome this limitation, it is planned to employ the nano-sustained release materials as a strategic solution to markedly prolong FGF4's therapeutic window and enhance its bioavailability. Fourth, while the current study employed a type 1 diabetes model, future research will use a type 2 diabetes model to evaluate the effects of FGF4. Fifth, the lack of an insulin control group *in vivo* also represents one of the limitations in the current study. Future research will focus on developing strategies to enhance the specific targeting of FGF4 to lung tissue, such as using lung-targeted nanomaterials.

FGF4 holds important significance in alleviating diabetic lung injury. Patients with diabetes often develop pulmonary complications and FGF4, as a novel long-acting glucose-regulating factor, can not only effectively regulate blood glucose but also alleviate pulmonary inflammation and injury through its anti-inflammatory and antioxidant properties. This discovery provides a new strategy for the treatment of diabetes-related lung injury, which is expected to improve the lung health of patients with diabetes, enhance their quality of life and holds important clinical and scientific value.

## Supplementary Data



## Data Availability

The data generated in the present study may be requested from the corresponding author.
