# Supplementary material for: FGF4 alleviates the lung cell damage caused by high glucose via AMPK-PGC-1 signaling axis in vitro and in vivo
Source: Int J Mol Med. 2025 Dec 5;57(2):39. doi: 10.3892/ijmm.2025.5710 (PMC12714404; doi:10.3892/ijmm.2025.5710)

Figure S1. Verification of AMPK knockdown efficiency by western blotting. AMPK, adenosine monophosphate activated protein kinase; si, short interfering.

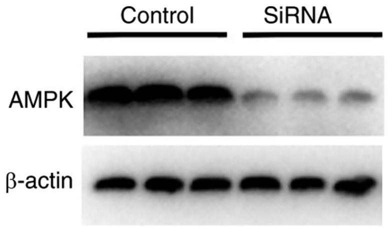

Figure S2. Detecting the effect of FGF4 on ROS and MDA in MLE12 and BEAS-2B. FGF4, fibroblast growth factor 4; ROS, reactive oxygen species; MDA, malondialdehyde. $^{*}P<0.05$, $^{**}P<0.01$, $^{***}P<0.001$.

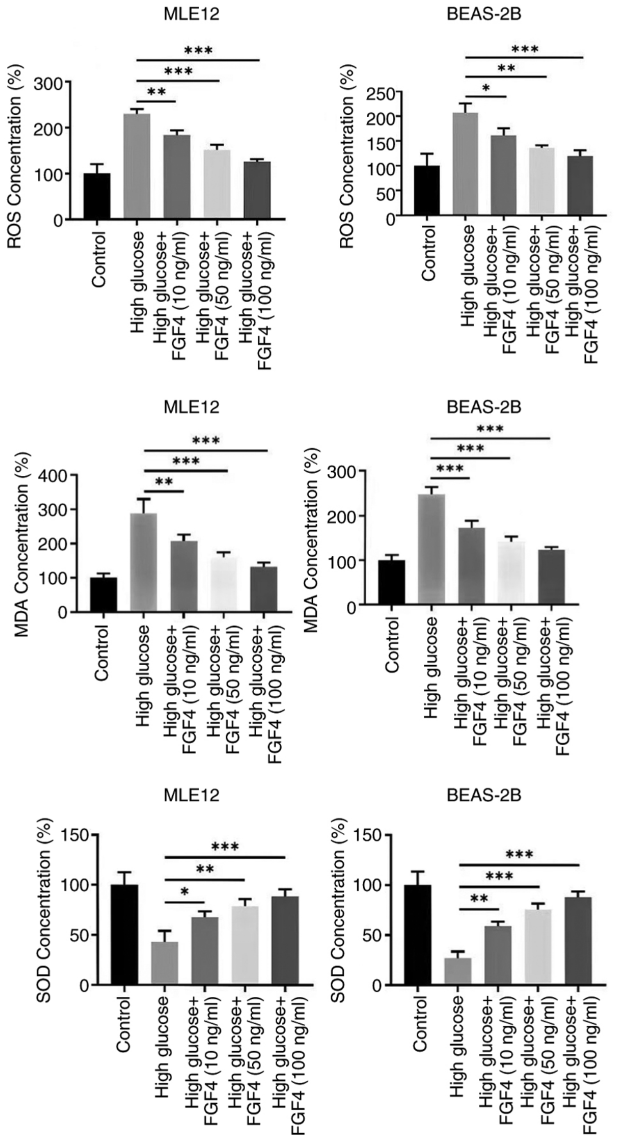

Figure S3. FGF4 regulates the expression of LC3II and TFAM through AMPK. FGF4, fibroblast growth factor 4; TFAM, mitochondrial transcription factor A; AMPK, adenosine monophosphate activated protein kinase.

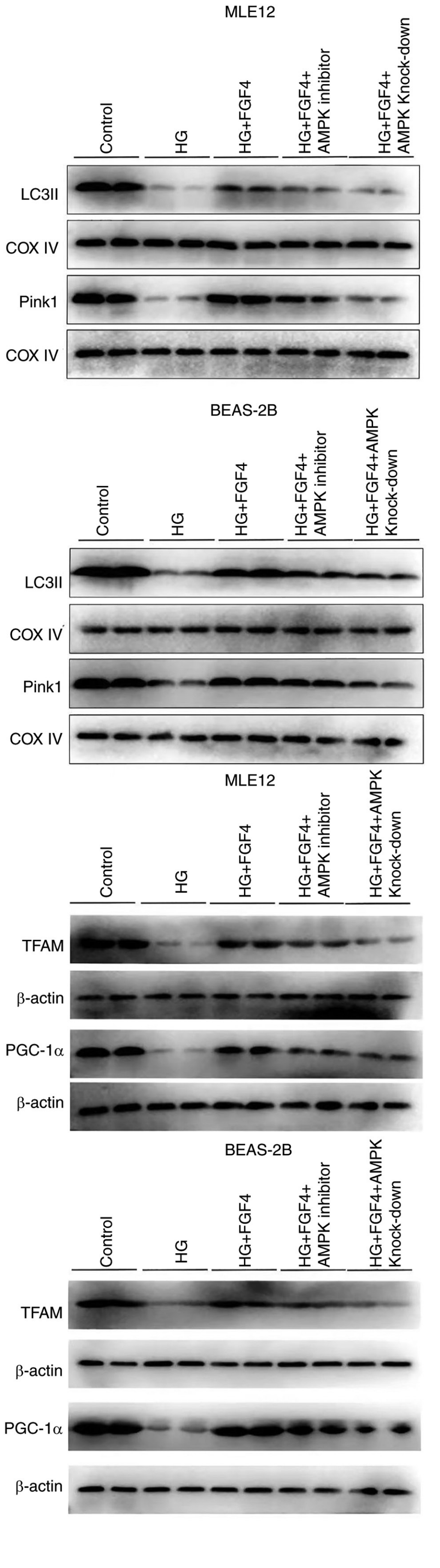

Figure S4. (A) The effect of FGF4 on the expression of TOM20 and TIM23. (B) FGF4 promoted the mitochondrial membrane potential. (C) The effect of FGF4 on the expression level of PINK1/Parkin. *P<0.05, **P<0.01, ***P<0.001. FGF4, fibroblast growth factor 4.

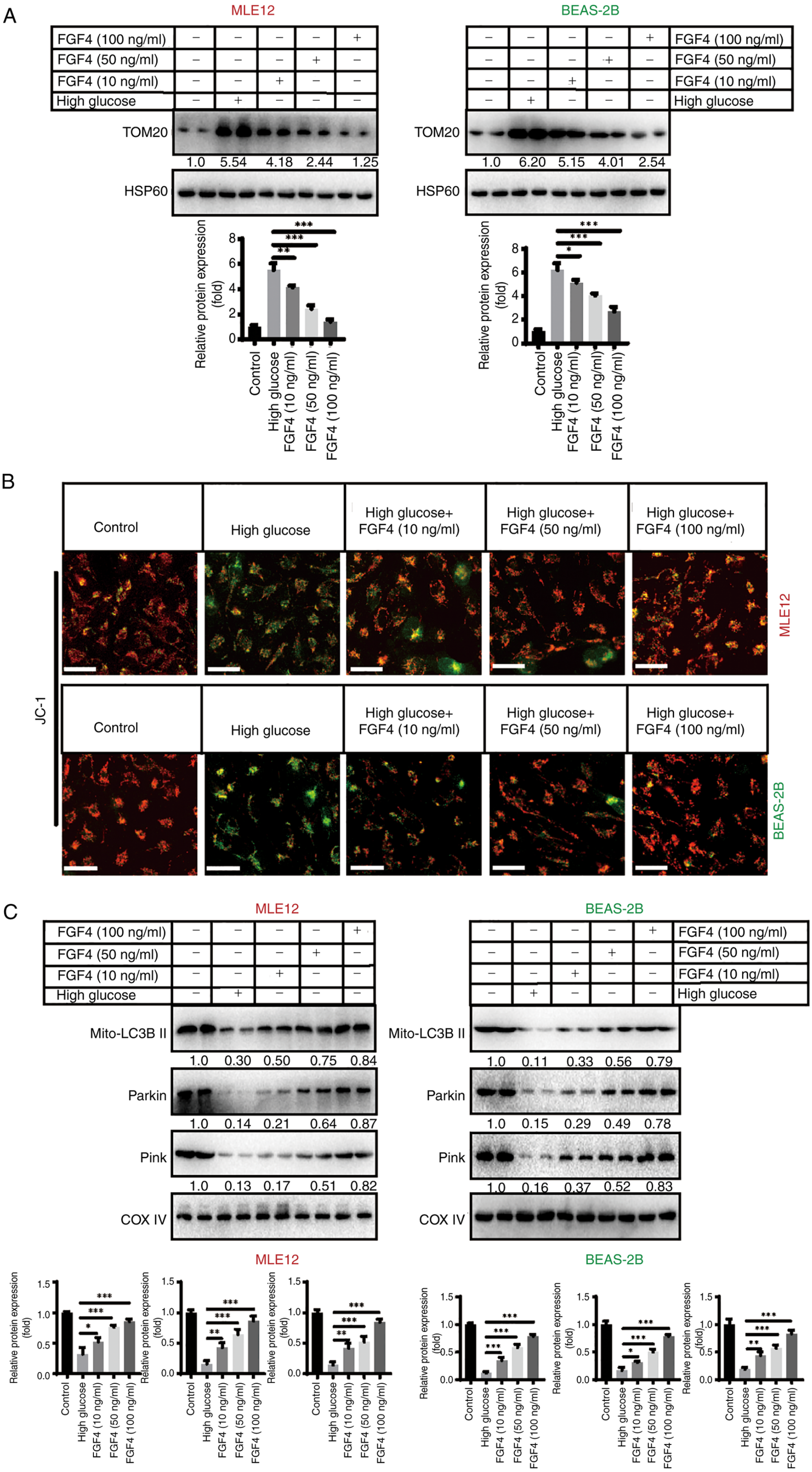

Supplement: Supplementary file 1 [file Supplementary_Data.pdf]
